# The Effects of Floods on the Incidence of Bacillary Dysentery in Baise (Guangxi Province, China) from 2004 to 2012

**DOI:** 10.3390/ijerph14020179

**Published:** 2017-02-12

**Authors:** Xuena Liu, Zhidong Liu, Ying Zhang, Baofa Jiang

**Affiliations:** 1 Department of Epidemiology and Health Statistics, School of Public Health, Shandong University, Jinan 250012, China; xuena_liu@163.com (X.L.); Liuzhidong3105@163.com (Z.L.); 2 Center for Climate Change and Health, School of Public Health, Shandong University, Jinan 250012, China; 3 School of Public Health, China Studies Centre, the University of Sydney, New South Wales 2006, Australia; ying.zhang@sydney.edu.au

**Keywords:** bacillary dysentery, floods, mixed generalized additive model, Spearman correlation

## Abstract

Research shows potential effects of floods on intestinal infections. Baise, a city in Guangxi Province (China) had experienced several floods between 2004 and 2012 due to heavy and constant precipitation. This study aimed to examine the relationship between floods and the incidence of bacillary dysentery in Baise. A mixed generalized additive model and Spearman correlation were applied to analyze the relationship between monthly incidence of bacillary dysentery and 14 flood events with two severity levels. Data collected from 2004 to 2010 were utilized to estimate the parameters, whereas data from 2011 to 2012 were used to validate the model. There were in total 9255 cases of bacillary dysentery included in our analyses. According to the mixed generalized additive model, the relative risks (RR) of moderate and severe floods on the incidence of bacillary dysentery were 1.40 (95% confidence interval (CI): 1.16–1.69) and 1.78 (95% CI: 1.61–1.97), respectively. The regression analysis also indicated that the flood duration was negatively associated with the incidence of bacillary dysentery (with RR: 0.57, 95% CI: 0.40–0.86). Therfore, this research suggests that floods exert a significant part in enhancing the risk of bacillary dysentery in Baise. Moreover, severe floods have a higher proportional contribution to the incidence of bacillary dysentery than moderate floods. In addition, short-term floods may contribute more to the incidence of bacillary dysentery than a long-term flood. The findings from this research will provide more evidence to reduce health risks related to floods.

## 1. Introduction

Floods are a common and serious type of natural hazard. On average, flood events account for 50% of the total number of natural disasters globally [1,2,3,4]. It is predicted that with more severe and frequent precipitation under future climatic scenarios, floods will increase in severity and frequency [5,6,7]. China is a country susceptible to various natural hazards, with many regions potentially affected by flooding [8]. Located in the tropical and subtropical monsoon region, Guangxi Province frequently suffers from floods [9].

The influences of floods are widespread and complicated, including increased number of deaths and incidence of diarrhea [7]. Floodwater can stimulate the growth of more pathogens, leading to a shortage of clean water. Many studies suggest that, due to floods after heavy rainfall, polluted drinking water is related with waterborne diseases and epidemics, including bacillary dysentery, typhoid fever and hepatitis A [10,11,12]. Bacillary dysentery, caused by *Shigella* bacteria, refers to a group of bacterial infections of the intestines, which may cause severe diarrhea. It is still a major public health problem around the world, particularly in developing countries [13]. According to the National Report of Notifiable Diseases from the Ministry of Health of China, bacillary dysentery is among the top three notified infectious diseases [14]. Though the Chinese government has developed a strategic plan for the supervision of prevention and control of bacillary dysentery at a national level, the number of cases of bacillary dysentery has continued to increase recently [2,15]. In the past decade, research has been conducted to examine the associations between weather and bacillary dysentery [14,16,17,18]. For example, several studies have reported that floods caused by heavy precipitation would be more likely to impact on death rates and the incidence of bacillary dysentery [2,19]. Moreover, the incidence of bacillary dysentery may increase due to the transmission of the pathogens after floods [20]. However, most previous studies explored the relationship between bacillary dysentery and floods with limited data from a single flood event. There is also a lack of analysis from a longitudinal perspective. 

There have been few studies conducted in southern China to examine the effects of various flood severity levels on bacillary dysentery [21]. Our study aims to quantify the association between the incidence of bacillary dysentery and the floods of various severity from 2004 to 2012 in Baise. Results will contribute to current knowledge of the health impacts of floods and assist in formulating effective local strategies to prevent and reduce the risk of bacterial dysentery related with floods.

## 2. Materials and Methods

### 2.1. Research Area

**Figure 1:**
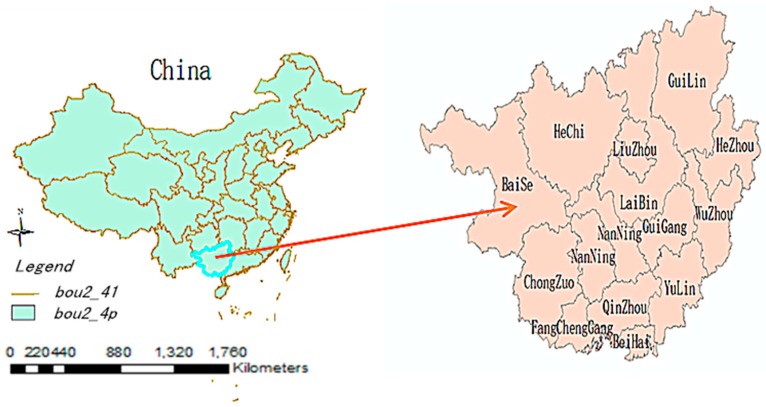
Location of Baise in Guangxi Province, China.

Located in the northwest region of Guangxi Province, Baise is an industrial and tourist destination city. It has a longitude of 104°28’E and 107°54’E, and latitude of 22°51’N and 25°07’N, covering an area of 36,000 km^2^ and hosting a population of 3,780,000 [22] (Figure 1). The Youjiang River, which is a tributary of the Pearl River, flows through Baise. The city has a subtropical monsoonal climate with an annual mean temperature ranging between 19.0 °C and 22.1 °C. The average precipitation is between 1113 mm and 1713 mm per year. From 2004 to 2012, severe and constant precipitation caused frequent floods in Baise, resulting in great economic losses and a high number of victims. This location thus provides an apt setting to explore the association between flooding and dysentery.

### 2.2. Data Collection and Management

#### 2.2.1. Disease Surveillance Data

This study collected monthly disease bacillary dysentery data between January 2004 to December 2012, from the National Notifiable Disease Surveillance System (NDSS). According to the NDSS, bacillary dysentery refers to a group of diseases resulting from Shigellae infection, with typical clinical manifestations such as stomach ache, fever, bloody stools and tenesmus. The occurrences of bacillary dysentery were identified in accordance with the diagnostic codes and management guidelines for bacillary dysentery (GB 16002-1995) promulgated by the Ministry of Health of China [23]. Only the cases identified by both biochemical identification and microscopy were included in this study. Listed as a statutory notifiable infectious disease in China, cases of bacillary dysentery must be reported to local health organizations. Subsequently, the local health organizations need to report to those to a higher level of health authority within 24 h [14]. The Direct Network Report system for infectious diseases, which was established by Chinese Center for Disease Control and Prevention, has been operated and managed systematically since 1 January 2004 with an accuracy rate close to 100% in recent years [24]. Therefore, the accuracy and reliability of the disease notification system had kept consistent over the study period.

#### 2.2.2. Floods Data

**Figure 2:**
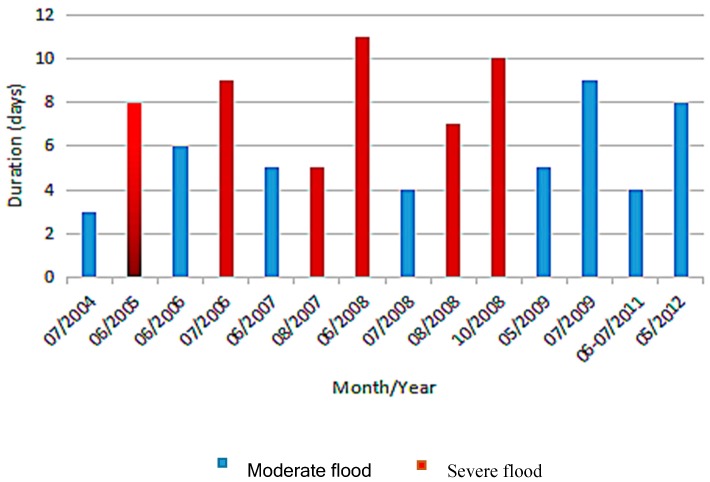
Floods recorded in the yearbook between 2004 and 2012 in Baise.

The yearbooks of meteorological disasters specifically recorded the incidence, mortality, disaster-stricken areas, and economic loss of flood events between 2004 and 2012 [25]. Based on the meteorological disaster yearbooks, a flood is defined as a natural hazard caused by the overflow of rivers because of sudden constant heavy rainfalls, leading to submerging of villages, lands, fatalities and economic losses. Floods with a fatality rate of 10–30 people or an economic loss of 1–3 billion yuan (approximately U.S.$ 16–48 million) are categorized as a moderate flood; while a severe flood usually causes over 30 fatalities or an economic loss of over 3 billion yuan (approximately US$ 48 million). There were 14 floods recorded in the yearbook between 2004 and 2012 in Baise, including eight moderate floods and six severe floods (Figure 2). In total, these fourteen floods affected 4,348,000 people and killed 19, while causing estimated economic damages of approximately 780 million yuan (U.S.$ 113 million).

#### 2.2.3. Meteorological and Demographic Data

The meteorological data were obtained from the China Meteorological Data Sharing Service System [26], which provided meteorological data from 752 weather stations over the period of 60 years. The meteorological variables included monthly cumulative precipitation (MCP, mm), monthly average temperature (MAT, °C), monthly average relative humidity (MARH, %), monthly average wind velocity (MAWV, m/s), and monthly cumulative sunshine duration (MCSD, h). A complete demographic data set was obtained from the Center for Public Health Science Data in China [27]. Table 1 shows the correlation analysis results of the meteorological factors.

### 2.3. Statistical Analysis

In our study, we used monthly data to examine the effects of floods on bacillary dysentery based on time-series data from 2004 to 2010, which included month with and without flooding events.

We firstly performed a descriptive analysis and the Mann–Whitney *U* test to illustrate the dissemination of the number of cases of bacillary dysentery and the climatic variables between the non-flooded and flooded months. Subsequently, Spearman's rank correlation was applied to evaluate the relationship between floods, climate factors, and the incidence of bacillary dysentery with different lagged values. The lagged value with the maximum correlation coefficient of each variable was then included in further regression analysis. Based on the latent period of bacillary dysentery as well as the living habits of the pathogen, a time lag between 0 to 2 months was taken into account [28].

Secondly, a mixed generalized additive model (MGAM) was established to quantify the association between various levels of floods and monthly incidence of bacillary dysentery, controlled for meteorological variables. Data between 2004 and 2010 were used to estimate the parameters, and data from 2011 and 2012 were used to validate the regression model. The result of the residual test indicated no overdispersion, and therefore, Poisson regression was used to quantify the association between various degrees of floods and monthly incidence of bacillary dysentery. A few studies revealed that meteorological factors, such as average relative dampness, average temperature, cumulative rainfalls, mean wind speed, and cumulative sunlight duration, were associated with diarrheal diseases [18,29,30]. Hence, these factors were controlled in the MGAM model. Generalized additive model (GAM) has been widely applied in time-series studies of the association between meteorological variables and health outcomes. The method can be used to model seasonality and long-term trend nonparametricallity, and it allows nonparametric and parametric functions to be studied jointly [31]. Considering the high correlation between floods and flood duration, this study examined floods and flood duration using two separate models to avoid collinearity. The regression models were described as follows.

Model 1:
  Ln (*Y_t_*) = Ln (*population*) + *β_0_* + *β_1_* (*floods*) + *s_1_* (*t*) + *s_2_* (*precipitation*) + *s_3_* (*temperature*) + *s_4_* (*humidity*) + *s_5_* (*wind velocity*) + *s_6_* (*sunshine duration*) + *s_7_* (sin2π*t*/12) (1)
Model 2:
  Ln (*Y_t_*) = Ln (*population*) + *β_0_* + *β_1_* (*duration*) + *s_1_* (*t*) + *s_2_* (*precipitation*) + *s_3_* (*temperature*) + *s_4_* (*humidity*) + *s_5_* (*wind velocity*) + *s_6_* (*sunshine duration*) + *s_7_* (sin2π*t*/12) (2)

where *Y_t_* is the monthly number of cases in time *t*. Ln (*population*) was used as an offset to make the model appropriate for rate data. *Floods* was coded as a categorical variable, with 0, 1, and 2 referring to the various levels of floods, i.e., non-floods, moderate floods, and severe floods. *Duration* denoted the number of days with flooding in a month. *s_2_* (*precipitation*), *s_3_* (*temperature*), *s_4_* (*humidity*), *s_5_* (*wind velocity*) and *s_6_* (*sunlight duration*) represent smooth functions of MCP, MAT, MARH, MAWV and MCSD, respectively, which were applied to adjust the impact of potential climate variable confounding. To control for potential long-term effects from demographic, social and economic development, and changes in health policy and services, the smooth spline of month was expressed as *s_1_* (*t*). Moreover, due to the seasonality of the disease -bacillary dysentery occurs more often in summer and autumn-, a sinusoidal term sin(2π*t*/12) was incorporated in the models to control seasonal variations [32]. SPSS 16.0 (SPSS Inc., Chicago, IL, USA) and R 3.1.3 (R Foundation for Statistical Computing, Vienna, Austria) were employed in all statistical analyses with a significance level of 0.05.

## 3. Results

### 3.1. Descriptive Analysis for the Disease and Meteorological Data

**Figure 3:**
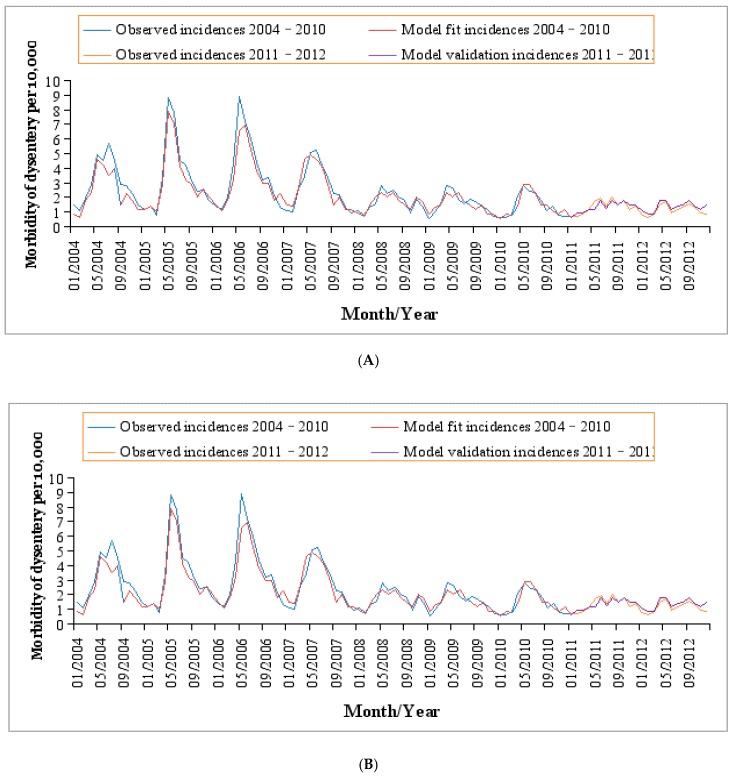
Observed cases vs. model fit and validation by the MGAM regression. (**A**) model 1 with adjusted R square was 0.90 and (**B**) model 2 with adjusted R square was 0.92.

In total, 9255 cases of bacillary dysentery were diagnosed in Baise during 2004–2012 with a mean annual incidence of 24.48/10,000. Table 2 demonstrates the distribution of the incidence of bacillary dysentery and climatic factors by month in the study region. Bacillary dysentery incidences, MCP, MAT, MARH and MCSD were significantly different between the non-flooded and flooded months (*p* < 0.05). Figure 3 shows the monthly bacillary dysentery incidence distribution of in Baise with a decreasing trend over the study period. Furthermore, a distinct seasonal trend was observed with more bacillary dysentery cases occurred in summer (June to August) and autumn (September to November).

### 3.2. Correlation Analysis

Table 3 demonstrates the results of Spearman’s correlation analysis. The monthly incidence of bacillary dysentery correlated positively with the moderate and severe floods, MCP, MAT, MARH and MCSD, with relevant lag times between 0 to 1 month. However, the flood duration and the MAWV correlated negatively with the incidence of bacillary dysentery, with a lag time of 0 and 1 month, respectively. The lagged effects of all meteorological factors were incorporated into further regression analysis.

Based on the results in Table 3, we examined lags 0 to 2 separately in a regression analysis. We found that only the 0 month (?) lag phase was statistically significant and had the maximum correlation coefficient. Given the correlation among both (?) lag phases which could lead to collinearity, we included only lag 0 in the following regression model.

### 3.3. Regression Analysis

The final parameters of the MGAM and relative risks (RRs) of different flood levels for the risk of bacillary dysentery are shown in Table 4. The observed monthly incidences of bacillary dysentery fitted well with the curves (?) predicted by the developed regression model, with a goodness of fit of 90% (adjusted R square *r_1_^2^* = 0.90) for model 1 and 92% (adjusted R square *r_2_^2^* = 0.92) for model 2 (Figure 3). Both moderate and severe floods were closely correlated with the incidence of bacillary dysentery (with coefficients: 0.34 for moderate floods and 0.58 for severe floods). Nevertheless, the flood duration was negatively correlated with bacillary dysentery incidences (with coefficient: −0.54). After controlling for the other meteorological variables, monthly bacillary dysentery incidences were positively correlated with the moderate floods (RR: 1.40, 95% confidence interval (CI): 1.16–1.69) and the severe floods (RR: 1.78, 95% CI: 1.61–1.97). A negative correlation existed between the floods duration and the incidence of bacillary dysentery (RR: 0.57, 95% CI: 0.40–0.86).

## 4. Discussion

Our study, quantified for the first time the relationship between various flood levels and bacillary dysentery incidences in Baise in Southern China. Results suggest that both moderate and severe floods would cause more bacillary dysentery cases in the research region, which is consistent with the findings in developed and developing countries. For instance, a study in Texas, USA, found that people in flooded areas were more likely to suffer from diarrhea than those in non-flooded areas during the floods in 2001 (with odds ratio (OR) = 10.8, *p* < 0.01) [33]. Another study conducted in Germany also suggested that contacting the floodwater was a major reason for diarrhea (with OR = 5.8, 95% CI = 1.3–25.1) [34]. A survey on impacts of the tropical storm Alison discovered that diarrhea was significantly correlated with people living in flooded areas (with OR = 6.2, 95% CI: 1.4–28.0) [35]. As for Lewes of Southern England, a study showed that people in the flooded area were significantly correlated with increased incidences of gastroenteritis during the floods in 2001 (RR: 1.7, *p* < 0.05) [36]. Moreover, our results are also consistent with some studies carried out in China. In Qingdao floods are positively associated with bacillary dysentery incidences (with RR = 1.42, 95% CI = 1.22–1.64) [37]. Another study showed that the RRs of floods on bacillary dysentery were 11.47 (with 95% CI: 8.67–15.33), 2.75 (with 95% CI: 1.36–4.85) and 1.35 (with 95% CI: 1.23–3.90) respectively in Kaifeng, Zhengzhou, and Xinxiang in Henan Province [38]. Compared to these studies conducted in Northern China, our study area is located in Southern China and has quite different climatic, social and economic conditions. Nevertheless, similar results were obtained. These findings indicate that floods could be an independent risk factor for bacillary dysentery incidences after the adjustment of potential confounders. 

Our study also identified that more (?) bacillary dysentery incidences may be caused by severe floods than by moderate ones. In Pakistan, a study showed that about 20% of the drinking water samples collected during flood periods were contaminated with *Shigella, Vibrio cholerae, Salmonella, Staphylococcus aureus*, and others. This means the risk of related water-borne disease may increase because of water contamination during flood periods [39]. There are several possible ways by which heavy rainfalls might influence the water pollution and increase the incidence risk of bacillary dysentery. First, excessive rainfall is more likely to cause the risk of overflows in sewers, which are used to move pathogens into lakes, wells, rivers, and seas. Worse still, it can unfavorably influence the water supply mechanism [40,41,42]. Second, more animal excreta and manure on the surface of soil or subsurface will be run off, which might form more pathogens in the surface of waters [43]. Third, more extreme rainfalls will increase disorders and cause sediment resuspension, scattering the accumulated pathogens [44,45,46]. Consequently, severe floods after extreme precipitation would worsen water quality via diverse means and lead to more chances for people to contact with flooded waters. In addition, some characteristics of Baise provide more chances for the spread of bacillary dysentery. For example, it has a beneficial temperature to the breed of dysentery bacterium. Baise is situated east of the YunGui plateau, and part of the region is highly mountainous with a large rural population. Food may be contaminated by contaminated water through the unhealthy life style of farmers, such as drinking unclear water or washing vegetables and fruits using dirty water. To our knowledge, the unique climatic condition in Baise and the life styles of local residents may be the most important contributors to the transmission of the pathogens.

The results of Model 2 indicate that a negative association exists between the floods duration and the incidence of bacillary dysentery. It indicates that the risk of suffering bacillary dysentery after a long-term flood may be lower than that after a short-term flood. At the early stage of the flood period after extreme rainfalls, the pathogens can develop and reproduce quickly in an appropriate environment, scattering through the polluted water and food [2]. Another study argued that the influences of enhanced or heavy rainfalls were short. With constant precipitation, the pathogens in the water may be diluted or flushed [44]. Some studies also demonstrate that short-term floods, with dramatically increased precipitation, may influence water supply systems, as well as sewerage and waste-disposal systems. This might lead to contaminated water sources and therefore increase the transmission of enteric pathogens during the floods, resulting in a higher chance of bacillary dysentery infection. Nonetheless, for a long-term flood, the infection of bacillary dysentery pathogens can be reduced because of lower pollution and destruction [47]. In spite of this, the existing studies have revealed different results. For instance, in Nanning (China), a study showed that the incidence of bacillary dysentery was higher after a long-term flood than after a shorter one after adjusting the meteorological factors and seasonal trend [21].

There are limitations in this study. Firstly, there are other variables, such as pathogen variability, population mobility, socioeconomic status, behavior and lifestyle changes, and environmental hygiene, that could impact the transmission of bacillary dysentery but could not be analyzed in this study. Secondly, the development of the disease from the exposure to diagnosis at hospitals up to being reported in the surveillance system may take 1–9 days. Therefore, more frequent data, e.g., daily or weekly incidences, of the disease, if available, would be more accurate than monthly data in our estimation of lag effects. Finally, underreporting is unavoidable in the research of infectious diseases, including bacillary dysentery. The reported cases included in this study are those who presented serious symptoms and were diagnosed in hospitals. Cases with mild clinical symptoms and treated by themselves usually did not seek health services, which may lead to an underestimate of the bacillary dysentery incidence risk by floods.

## 5. Conclusions

In conclusion, our study suggests that floods can greatly increase the risk of bacillary dysentery, particularly severe floods. Moreover, a short-term flood may lead to more incidences of bacillary dysentery than a long-term flood event if the floods are at the same severity level. The findings of this study will provide more evidence to reduce health risks of floods in China.

## Figures and Tables

**Table 1:** The results of correlation analysis of the meteorological factors.

Meteorological Factors	MCP	MAT	MARH	MAWV	MCSD
MCP	1.000				
MAT	−0.236 *	1.000			
MARH	0.437 *	−0.214 *	1.000		
MAWV	−0.070	0.393 *	−0.211 *	1.000	
MCSD	−0.389 *	0.554 *	−0.565 *	0.162 *	1.000

* *p* < 0.05. MCP: monthly cumulative precipitation; MAT: monthly average temperature; MARH: monthly average relative humidity; MAWV: monthly average wind velocity; MCSD: monthly cumulative sunshine duration.

**Table 2:** Description of the incidence of bacillary dysentery and climate variables from 2004 to 2010 in Baise.

Analyzed Variables	Flooded Months	Mean ± SD	Min	P_25_	Median	P_75_	Max
Incidence of bacillary dysentery	Yes *	133 ± 90	35	66	94	221	296
	No	79 ± 57	20	42	59	102	338
MCP (mm)	Yes *	7.54 ± 17.88	0.00	0.00	0.10	6.05	134.10
	No	2.30 ± 8.19	0.00	0.00	0.00	0.20	113.30
MAT (°C)	Yes *	27.35 ± 2.23	20.30	25.90	27.60	28.90	32.80
	No	21.51 ± 6.37	4.70	16.30	22.60	27.00	32.70
MARH (%)	Yes *	77.78 ± 8.87	45.00	72.00	78.00	84.00	100.00
	No	72.98 ± 10.56	25.00	66.75	73.00	80.00	100.00
MAWV (m/s)	Yes	1.48 ± 0.54	0.50	1.10	1.40	1.80	3.70
	No	1.43 ± 0.61	0.00	1.00	1.30	1.80	4.50
MCSD (h)	Yes *	4.58 ± 3.57	0.00	1.10	4.50	7.60	11.60
	No	4.30 ± 3.76	0.00	0.00	4.20	7.70	11.80

* *p* < 0.05 vs. non-flooded month. SD: standard deviation; Min: minimum; P_25_: the 25th percentile; P_75_: the 75th percentile; Max: maximum

**Table 3:** Spearman’s rank correlations between the incidence of bacillary dysentery and explanatory variables among monthly data in Baise from 2004 to 2010.

Variables	Lag 0	Lag 1	Lag 2
	R (*p* Value)	R (*p* Value)	R (*p* Value)
Floods	0.51 (<0.01)	0.42 (0.05)	0.38 (0.12)
Duration (days)	−0.41 (0.01)	−0.30 (0.08)	−0.30 (0.06)
MCP (mm)	0.67 (<0.01)	0.61 (<0.01)	0.57 (<0.01)
MAT (°C)	0.49 (<0.01)	0.43 (<0.01)	0.39 (<0.01)
MARH (%)	0.25 (0.02)	0.21 (0.23)	0.23 (0.17)
MAWV (m/s)	−0.28 (0.11)	−0.34 (0.01)	−0.21 (0.24)
MCSD (h)	0.29 (<0.01)	0.36 (<0.01)	0.24 (0.37)

**Table 4:** Parameters estimated by the MGAM for bacillary dysentery in Baise.

Model	Variables	Coefficients	*p* Value	RR (95% CI)
Model 1 *	Moderate floods	0.34	<0.01	1.40 (1.16–1.69)
	Severe floods	0.58	<0.01	1.78 (1.61–1.97)
	Reference (no flood)	-	-	-
Model 2 *	Duration	−0.54	<0.01	0.57 (0.40–0.86)

* Adjusted R square was 0.90 for model 1 and 0.92 for model 2. CI: confidence interval; MGAM: mixed generalized additive model; RR: relative risk.
